# Supplementary material for: Male and Female Perceptions of Supervision During Strength Training
Source: Sports (Basel). 2024 Nov 5;12(11):301. doi: 10.3390/sports12110301 (PMC11598539; doi:10.3390/sports12110301)
Supplement: Supplementary file 1 [file sports-12-00301-s001.zip › sports-3285544-supplementary.pdf]

Perceptions of supervision during Strength Training

Survey is Open

Once you have launched your survey you will be prevented from making changes that may affect response data already collected. Please see our help page on making changes to open surveys, which can be found here.

When creating your survey, we recommend the use of a privacy notice, this should explain to survey respondents about how you plan to use any personal information you collect, and how long you intend on keeping it. Your organisation's data protection officer may be able to provide advice and guidance on creating a suitable privacy notice for your survey.

p. 1 Page 1

Add item

Your Consent

You are invited to take part in a study exploring your perceptions of supervision during strength training. Whether or not you take part is your choice. If you do want to take part now, but change your mind later, you can leave the study at any time.

Why are we doing the study?
Strength training provides numerous health benefits, however global participation in strength training is low (5-30%). Asking persons currently involved in strength training about their perceptions of supervision might provide information which can promote engagement in strength training practices.

What is involved?
The study will involve the completion of a short online questionnaire about your strength training. This questionnaire should take ~5 minutes in total to complete.

Are there any risks to being part of this study?
The risks involved in the study are low, although we acknowledge thinking about your behaviours, health and well-being may be sensitive topics to some people.

What are the rights of participants in the study?
Taking part in this study is voluntary. You are free to say no to participating, or to leave the research study at any time, without any disadvantage. All data will be handled in compliance with the General Data Protection Regulation which came into law on May 25th 2018. You have the right to access any information that we obtain on you from the study. Data will be stored securely in password protected files on a secure cloud server. Only the research team who are directly involved in the study will have access to identifiable data. The anonymised dataset from the study will be stored in a publically available server to facilitate sharing of data with other researchers who may be able to utilise the data for relevant research. Any data shared publically will be non-identifiable. Aggregated results will be presented in public reports including pre-print and open access scientific articles.

Ethical Approval

This study received ethical approval from the Health, Exercise, and Sport Science (HESS) Ethics Committee at Solent University (fishj1HESS2022).

Who are the investigators?
Dr James Fisher, Solent University, Southampton, UK,

Contact details of lead investigator: james.fisher@solent.ac.uk

By clicking continue you are consenting that have read and understand the above information pertaining to participation in this study. You understand that participation is voluntary and that you are free to withdraw at any time, without giving any reason, without treatment or legal rights being affected. You agree to the secure storage of data, in line with the General Data Protection Regulation, as it pertains to the above study, and are aware that your identity will remain confidential.

Add item

p. 2 Demographic details

Add item

1 What is your age?

<20 years

20-29 years

30-39 years

Show all (7)

Add item

Add item

2 What is the biological sex to which you were assigned at birth?

Male

Female

Add item

Add item

3 For how many years have you been engaging in supervised strength training?

<1 year

1-2 years

2-3 years

Show all (5)

Add item

Add item

4 What is your primary reason for engaging in strength training? *

Sporting/athletic performance

Health improvements (including stress relief, weight loss, etc.)

Ill health avoidance

Show all (6)

Add item

Add item

p. 3 Effects of Supervision

Add item

5 Please rate the importance of supervision for these characteristics of strength training: *

	Not important	Somewhat important	Modestly important	Very Important	Essential
Technical - Correcting/maintaining your form, providing technical feedback on performance:	☐	☐	☐	☐	☐
Effort - Increasing/maintaining your effort level, including use of forced repetitions, negative repetitions, breakdown sets, etc. to exercise beyond failure:	☐	☐	☐	☐	☐
Program Design/Workout Routine - Exercise choice, load, progression, etc:	☐	☐	☐	☐	☐
Motivational - Providing encouragement to complete a workout, to promote enjoyment, etc:	☐	☐	☐	☐	☐
Accountability - Promoting engagement and adherence:	☐	☐	☐	☐	☐
Safety - Spotting, technique correction to prevent injury, handing weights, etc:	☐	☐	☐	☐	☐

Add item

Add item

p. 4 Technique

Add item

6 Please rate the importance of supervision for technique during strength training: *

	Strongly Disagree	Disagree	Neither Agree nor Disagree	Agree	Strongly Agree
My technique is good because my strength training workouts are supervised:	☐	☐	☐	☐	☐
My technique would always be good even without supervision:	☐	☐	☐	☐	☐
I value feedback about my technique from a personal trainer:	☐	☐	☐	☐	☐
I dislike it when my personal trainer corrects my technique:	☐	☐	☐	☐	☐
Technique during strength training is impactful in my ability to meet my desired goals:	☐	☐	☐	☐	☐

Add item

Add item

p. 5 Effort

Add item

7 Please rate the importance of supervision for effort during strength training: *

	Strongly Disagree	Disagree	Neither Agree nor Disagree	Agree	Strongly Agree
My effort level is highest because my strength training workouts are supervised:	☐	☐	☐	☐	☐
My effort level would always be high even without supervision:	☐	☐	☐	☐	☐
I value a personal trainer pushing me to work harder:	☐	☐	☐	☐	☐
I dislike it when my personal trainer pushes me too hard:	☐	☐	☐	☐	☐
Effort during strength training is impactful in my ability to meet my desired goals:	☐	☐	☐	☐	☐

Add item

Add item

p. 6 Program Design/Workout Routine

Add item

8 Please rate the importance of program design during strength training: *

	Strongly Disagree	Disagree	Neither Agree nor Disagree	Agree	Strongly Agree
My strength training workouts are better because of programming by a personal trainer:	☐	☐	☐	☐	☐
I would feel confident to track and program my own workouts without a personal trainer:	☐	☐	☐	☐	☐
I value my personal trainer tracking and progressing my workouts over time:	☐	☐	☐	☐	☐
I dislike it when my personal trainer progresses my load/changes exercises without discussion:	☐	☐	☐	☐	☐
The strength training program I use is impactful in my ability to meet my goals:	☐	☐	☐	☐	☐

Add item

Add item

p. 7 Motivation

Add item

9 Please rate the importance of motivation during strength training: *

	Strongly Disagree	Disagree	Neither Agree nor Disagree	Agree	Strongly Agree
My strength training workouts are more enjoyable because of supervision by a personal trainer:	☐	☐	☐	☐	☐
I would enjoy my workouts to a similar extent without supervision:	☐	☐	☐	☐	☐
I value a personal trainer encouraging me with positive feedback:	☐	☐	☐	☐	☐
I dislike it when my personal trainer tries to motivate me:	☐	☐	☐	☐	☐
My enjoyment of strength training is impactful in my ability to meet my goals:	☐	☐	☐	☐	☐

Add item

Add item

p. 8 Accountability

Add item

10 Please rate the importance of accountability during strength training: *

	Strongly Disagree	Disagree	Neither Agree nor Disagree	Agree	Strongly Agree
My strength training workouts are more frequent because of supervision by a personal trainer:	☐	☐	☐	☐	☐
My workouts would have similar frequency if I were not meeting a personal trainer:	☐	☐	☐	☐	☐
I value the accountability of meeting personal trainer for strength training workouts:	☐	☐	☐	☐	☐
I dislike having to schedule strength training workouts:	☐	☐	☐	☐	☐
The regularity of my strength training is impactful in my ability to meet my goals:	☐	☐	☐	☐	☐

Add item

Add item

p. 9 Safety

Add item

11 Please rate the importance of safety during strength training: *

	Strongly Disagree	Disagree	Neither Agree nor Disagree	Agree	Strongly Agree
My strength training workouts are safer because of supervision by a personal trainer:	☐	☐	☐	☐	☐
My workouts would be equally safe even if I were not supervised a personal trainer:	☐	☐	☐	☐	☐
I value the safety of having a personal trainer present for my strength training workouts:	☐	☐	☐	☐	☐
My strength training workouts are too focused on safety:	☐	☐	☐	☐	☐
The safety of my strength training is impactful in my ability to meet my goals:	☐	☐	☐	☐	☐

Add item

Add item

p. 10 Other

Add item

12 Using the same scale please answer these final few questions: *

	Strongly Disagree	Disagree	Neither Agree nor Disagree	Agree	Strongly Agree
Supervision by a Personal Trainer optimizes strength training adaptations:	☐	☐	☐	☐	☐
Supervision by a Personal Trainer optimizes efficiency of a strength training workout:	☐	☐	☐	☐	☐
Supervision by a Personal Trainer reduces anxiety over performing strength training:	☐	☐	☐	☐	☐
Supervision by a Personal Trainer improves confidence when performing strength training:	☐	☐	☐	☐	☐
I have a more positive attitude toward strength training because of supervision by a personal trainer:	☐	☐	☐	☐	☐
Feedback from a personal trainer is an important part of my strength training:	☐	☐	☐	☐	☐
I have confidence in my personal trainer because of their experience:	☐	☐	☐	☐	☐
I have confidence in my personal trainer because of their qualifications:	☐	☐	☐	☐	☐
I enjoy working with a team of different personal trainers:	☐	☐	☐	☐	☐

Add item

Add item

p. 11 Other comments

Add item

13 Please share any other thoughts around supervised strength training here:

Add item

Add item

p. 12 Qualitative follow-up

Add item

14 If you would be open to a follow-up discussion about supervision during your strength training, please insert your email address below.

Add item

Add item

p. 13 Final page

Add item

Thank you for your time completing this survey.

Should you have any final comments, please contact the lead investigator:

Dr. James Fisher

Solent University, UK

email: james.fisher@solent.ac.uk

Add item

Perceptions of supervision during Strength Training

Cross tabulation created ×

Showing 369 of 369 responses

Showing **all** responses

Hiding questions **2**, **13** & **14**

Response rate: 369%

1. What is your age?

What is your age?	What is the biological sex to which you were assigned at birth?		No answer
	Male	Female	
<20 years	0.00%	0.00%	0.00%
20-29 years	2.99%	3.88%	0.00%
30-39 years	6.72%	11.64%	33.33%
40-49 years	31.34%	17.24%	0.00%
50-59 years	18.66%	27.16%	66.67%
60-69 years	29.85%	28.02%	0.00%
70+ years	10.45%	12.07%	0.00%
No answer	0.00%	0.00%	0.00%

Question	Response count
2	366
1	369

3. For how many years have you been engaging in supervised strength training?

For how many years have you been engaging in supervised strength training?	What is the biological sex to which you were assigned at birth?		No answer
	Male	Female	
<1 year	9.70%	18.53%	0.00%
1-2 years	13.43%	15.52%	0.00%
2-3 years	11.94%	14.66%	33.33%
3-4 years	10.45%	8.19%	0.00%
5+ years	54.48%	42.67%	66.67%
No answer	0.00%	0.43%	0.00%

Question	Response count
2	366
3	368

4. What is your primary reason for engaging in strength training?

What is your primary reason for engaging in strength training?	What is the biological sex to which you were assigned at birth?		No answer
	Male	Female	
Sporting/athletic performance	5.97%	4.31%	66.67%
Health improvements (including stress relief, weight loss, etc.)	52.99%	51.29%	0.00%
Ill health avoidance	8.21%	9.91%	0.00%
Social reasons	0.00%	0.00%	0.00%
Aesthetics (e.g., muscle size)	1.49%	0.43%	33.33%
Physical function other than sports performance (e.g., muscle strength for day to day activities)	31.34%	34.05%	0.00%
No answer	0.00%	0.00%	0.00%

Question	Response count
2	366
4	369

5.1. Technical - Correcting/maintaining your form, providing technical feedback on performance:

Technical - Correcting/maintaining your form, providing technical feedback on performance:	What is the biological sex to which you were assigned at birth?		No answer
	Male	Female	
Not important	0.00%	0.00%	0.00%
Somewhat important	0.00%	0.86%	0.00%
Modestly important	10.45%	2.59%	0.00%
Very Important	39.55%	21.98%	66.67%
Essential	50.00%	74.57%	33.33%
No answer	0.00%	0.00%	0.00%

Question	Response count
2	366
5.1	369

5.2. Effort - Increasing/maintaining your effort level, including use of forced repetitions, negative repetitions, breakdown sets, etc. to exercise beyond failure:

Effort - Increasing/maintaining your effort level, including use of forced repetitions, negative repetitions, breakdown sets, etc. to exercise beyond failure:	What is the biological sex to which you were assigned at birth?		No answer
	Male	Female	
Not important	0.00%	0.43%	0.00%
Somewhat important	1.49%	2.16%	0.00%
Modestly important	4.48%	6.03%	33.33%
Very Important	32.09%	31.47%	33.33%
Essential	61.94%	59.91%	33.33%
No answer	0.00%	0.00%	0.00%

Question	Response count
2	366
5.2	369

5.3. Program Design/Workout Routine - Exercise choice, load, progression, etc:

Program Design/Workout Routine - Exercise choice, load, progression, etc:	What is the biological sex to which you were assigned at birth?		No answer
	Male	Female	
Not important	0.00%	0.00%	0.00%
Somewhat important	1.49%	0.86%	0.00%
Modestly important	11.94%	8.19%	33.33%
Very Important	46.27%	35.34%	33.33%
Essential	40.30%	55.60%	33.33%
No answer	0.00%	0.00%	0.00%

Question	Response count
2	366
5.3	369

5.4. Motivational - Providing encouragement to complete a workout, to promote enjoyment, etc:

Motivational - Providing encouragement to complete a workout, to promote enjoyment, etc:	What is the biological sex to which you were assigned at birth?		No answer
	Male	Female	
Not important	1.49%	2.16%	0.00%
Somewhat important	4.48%	2.59%	0.00%
Modestly important	19.40%	10.34%	33.33%
Very Important	38.81%	45.69%	33.33%
Essential	35.82%	39.22%	33.33%
No answer	0.00%	0.00%	0.00%

Question	Response count
2	366
5.4	369

5.5. Accountability - Promoting engagement and adherence:

Accountability - Promoting engagement and adherence:	What is the biological sex to which you were assigned at birth?		No answer
	Male	Female	
**Not important**	0.75%	2.59%	0.00%
**Somewhat important**	7.46%	3.45%	0.00%
**Modestly important**	12.69%	19.40%	33.33%
**Very Important**	42.54%	34.48%	0.00%
**Essential**	36.57%	40.09%	66.67%
**No answer**	0.00%	0.00%	0.00%

Question	Response count
**2**	366
**5.5**	369

5.6. Safety - Spotting, technique correction to prevent injury, handing weights, etc:

Safety - Spotting, technique correction to prevent injury, handing weights, etc:	What is the biological sex to which you were assigned at birth?		No answer
	Male	Female	
**Not important**	1.49%	0.43%	0.00%
**Somewhat important**	8.96%	1.29%	33.33%
**Modestly important**	11.94%	6.03%	33.33%
**Very Important**	29.85%	20.69%	0.00%
**Essential**	47.76%	71.55%	33.33%
**No answer**	0.00%	0.00%	0.00%

Question	Response count
**2**	366
**5.6**	369

6.1. My technique is good because my strength training workouts are supervised:

My technique is good because my strength training workouts are supervised:	What is the biological sex to which you were assigned at birth?		No answer
	Male	Female	
Strongly Disagree	0.00%	0.43%	0.00%
Disagree	0.00%	0.86%	0.00%
Neither Agree nor Disagree	4.48%	3.02%	0.00%
Agree	35.82%	21.98%	33.33%
Strongly Agree	59.70%	73.71%	66.67%
No answer	0.00%	0.00%	0.00%

Question	Response count
2	366
6.1	369

6.2. My technique would always be good even without supervision:

My technique would always be good even without supervision:	What is the biological sex to which you were assigned at birth?		No answer
	Male	Female	
Strongly Disagree	13.43%	16.81%	0.00%
Disagree	48.51%	53.02%	0.00%
Neither Agree nor Disagree	20.90%	18.10%	66.67%
Agree	15.67%	9.48%	0.00%
Strongly Agree	1.49%	2.59%	33.33%
No answer	0.00%	0.00%	0.00%

Question	Response count
2	366
6.2	369

6.3. I value feedback about my technique from a personal trainer:

I value feedback about my technique from a personal trainer:	What is the biological sex to which you were assigned at birth?		No answer
	Male	Female	
Strongly Disagree	0.00%	0.00%	0.00%
Disagree	0.00%	0.00%	0.00%
Neither Agree nor Disagree	0.75%	0.86%	0.00%
Agree	29.10%	19.40%	66.67%
Strongly Agree	70.15%	79.74%	33.33%
No answer	0.00%	0.00%	0.00%

Question	Response count
2	366
6.3	369

6.4. I dislike it when my personal trainer corrects my technique:

I dislike it when my personal trainer corrects my technique:	What is the biological sex to which you were assigned at birth?		No answer
	Male	Female	
Strongly Disagree	66.42%	76.72%	0.00%
Disagree	27.61%	19.40%	66.67%
Neither Agree nor Disagree	5.22%	1.29%	0.00%
Agree	0.75%	1.29%	0.00%
Strongly Agree	0.00%	1.29%	33.33%
No answer	0.00%	0.00%	0.00%

Question	Response count
2	366
6.4	369

6.5. Technique during strength training is impactful in my ability to meet my desired goals:

Technique during strength training is impactful in my ability to meet my desired goals:	What is the biological sex to which you were assigned at birth?		No answer
	Male	Female	
Strongly Disagree	0.75%	0.00%	0.00%
Disagree	2.24%	0.43%	0.00%
Neither Agree nor Disagree	2.99%	3.02%	33.33%
Agree	39.55%	28.88%	33.33%
Strongly Agree	54.48%	67.67%	33.33%
No answer	0.00%	0.00%	0.00%

Question	Response count
2	366
6.5	369

7.1. My effort level is highest because my strength training workouts are supervised:

My effort level is highest because my strength training workouts are supervised:	What is the biological sex to which you were assigned at birth?		No answer
	Male	Female	
Strongly Disagree	0.75%	0.43%	0.00%
Disagree	1.49%	1.72%	0.00%
Neither Agree nor Disagree	2.99%	3.02%	0.00%
Agree	30.60%	24.57%	0.00%
Strongly Agree	64.18%	70.26%	100.00%
No answer	0.00%	0.00%	0.00%

Question	Response count
2	366
7.1	369

7.2. My effort level would always be high even without supervision:

My effort level would always be high even without supervision:	What is the biological sex to which you were assigned at birth?		No answer
	Male	Female	
Strongly Disagree	13.43%	18.53%	33.33%
Disagree	44.78%	50.00%	33.33%
Neither Agree nor Disagree	19.40%	14.22%	33.33%
Agree	19.40%	15.09%	0.00%
Strongly Agree	2.99%	2.16%	0.00%
No answer	0.00%	0.00%	0.00%

Question	Response count
2	366
7.2	369

7.3. I value a personal trainer pushing me to work harder:

I value a personal trainer pushing me to work harder:	What is the biological sex to which you were assigned at birth?		No answer
	Male	Female	
Strongly Disagree	0.00%	1.72%	0.00%
Disagree	0.75%	0.43%	0.00%
Neither Agree nor Disagree	3.73%	3.02%	0.00%
Agree	26.87%	24.57%	0.00%
Strongly Agree	68.66%	70.26%	100.00%
No answer	0.00%	0.00%	0.00%

Question	Response count
2	366
7.3	369

7.4. I dislike it when my personal trainer pushes me too hard:

I dislike it when my personal trainer pushes me too hard:	What is the biological sex to which you were assigned at birth?		No answer
	Male	Female	
**Strongly Disagree**	47.76%	40.52%	66.67%
**Disagree**	33.58%	30.17%	0.00%
**Neither Agree nor Disagree**	11.19%	13.36%	0.00%
**Agree**	7.46%	12.07%	33.33%
**Strongly Agree**	0.00%	3.88%	0.00%
**No answer**	0.00%	0.00%	0.00%

Question	Response count
2	366
7.4	369

7.5. Effort during strength training is impactful in my ability to meet my desired goals:

Effort during strength training is impactful in my ability to meet my desired goals:	What is the biological sex to which you were assigned at birth?		No answer
	Male	Female	
**Strongly Disagree**	0.00%	0.43%	0.00%
**Disagree**	0.00%	0.86%	0.00%
**Neither Agree nor Disagree**	0.75%	0.86%	0.00%
**Agree**	26.12%	25.00%	33.33%
**Strongly Agree**	73.13%	72.84%	66.67%
**No answer**	0.00%	0.00%	0.00%

Question	Response count
2	366
7.5	369

8.1. My strength training workouts are better because of programming by a personal trainer:

My strength training workouts are better because of programming by a personal trainer:	What is the biological sex to which you were assigned at birth?		No answer
	Male	Female	
Strongly Disagree	0.00%	0.00%	0.00%
Disagree	0.00%	0.00%	0.00%
Neither Agree nor Disagree	2.99%	2.59%	0.00%
Agree	27.61%	18.10%	66.67%
Strongly Agree	69.40%	79.31%	33.33%
No answer	0.00%	0.00%	0.00%

Question	Response count
2	366
8.1	369

8.2. I would feel confident to track and program my own workouts without a personal trainer:

I would feel confident to track and program my own workouts without a personal trainer:	What is the biological sex to which you were assigned at birth?		No answer
	Male	Female	
Strongly Disagree	14.18%	36.21%	0.00%
Disagree	44.03%	43.10%	66.67%
Neither Agree nor Disagree	22.39%	14.22%	0.00%
Agree	14.18%	5.17%	0.00%
Strongly Agree	5.22%	1.29%	33.33%
No answer	0.00%	0.00%	0.00%

Question	Response count
2	366
8.2	369

8.3. I value my personal trainer tracking and progressing my workouts over time:

I value my personal trainer tracking and progressing my workouts over time:	What is the biological sex to which you were assigned at birth?		No answer
	Male	Female	
**Strongly Disagree**	0.00%	0.00%	0.00%
**Disagree**	0.00%	0.43%	0.00%
**Neither Agree nor Disagree**	4.48%	0.86%	0.00%
**Agree**	36.57%	30.17%	66.67%
**Strongly Agree**	58.96%	68.53%	33.33%
**No answer**	0.00%	0.00%	0.00%

Question	Response count
**2**	366
**8.3**	369

8.4. I dislike it when my personal trainer progresses my load/changes exercises without discussion:

I dislike it when my personal trainer progresses my load/changes exercises without discussion:	What is the biological sex to which you were assigned at birth?		No answer
	Male	Female	
**Strongly Disagree**	39.55%	37.07%	33.33%
**Disagree**	36.57%	40.95%	66.67%
**Neither Agree nor Disagree**	14.18%	13.79%	0.00%
**Agree**	8.21%	6.03%	0.00%
**Strongly Agree**	1.49%	2.16%	0.00%
**No answer**	0.00%	0.00%	0.00%

Question	Response count
**2**	366
**8.4**	369

8.5. The strength training program I use is impactful in my ability to meet my goals:

The strength training program I use is impactful in my ability to meet my goals:	What is the biological sex to which you were assigned at birth?		No answer
	Male	Female	
Strongly Disagree	0.00%	0.43%	0.00%
Disagree	0.00%	0.43%	0.00%
Neither Agree nor Disagree	0.75%	2.16%	0.00%
Agree	29.85%	28.45%	66.67%
Strongly Agree	69.40%	68.53%	33.33%
No answer	0.00%	0.00%	0.00%

Question	Response count
2	366
8.5	369

9.1. My strength training workouts are more enjoyable because of supervision by a personal trainer:

My strength training workouts are more enjoyable because of supervision by a personal trainer:	What is the biological sex to which you were assigned at birth?		No answer
	Male	Female	
Strongly Disagree	0.00%	0.00%	0.00%
Disagree	0.00%	0.00%	0.00%
Neither Agree nor Disagree	5.22%	6.47%	0.00%
Agree	32.09%	25.43%	33.33%
Strongly Agree	62.69%	68.10%	66.67%
No answer	0.00%	0.00%	0.00%

Question	Response count
2	366
9.1	369

9.2. I would enjoy my workouts to a similar extent without supervision:

I would enjoy my workouts to a similar extent without supervision:	What is the biological sex to which you were assigned at birth?		No answer
	Male	Female	
Strongly Disagree	17.16%	30.60%	33.33%
Disagree	56.72%	47.84%	66.67%
Neither Agree nor Disagree	17.16%	11.64%	0.00%
Agree	8.21%	8.62%	0.00%
Strongly Agree	0.75%	1.29%	0.00%
No answer	0.00%	0.00%	0.00%

Question	Response count
2	366
9.2	369

9.3. I value a personal trainer encouraging me with positive feedback:

I value a personal trainer encouraging me with positive feedback:	What is the biological sex to which you were assigned at birth?		No answer
	Male	Female	
Strongly Disagree	0.00%	0.43%	0.00%
Disagree	0.00%	0.00%	0.00%
Neither Agree nor Disagree	2.24%	2.16%	0.00%
Agree	38.81%	28.45%	33.33%
Strongly Agree	58.96%	68.97%	66.67%
No answer	0.00%	0.00%	0.00%

Question	Response count
2	366
9.3	369

9.4. I dislike it when my personal trainer tries to motivate me:

I dislike it when my personal trainer tries to motivate me:	What is the biological sex to which you were assigned at birth?		No answer
	Male	Female	
Strongly Disagree	55.97%	54.74%	33.33%
Disagree	38.06%	37.50%	66.67%
Neither Agree nor Disagree	5.97%	3.45%	0.00%
Agree	0.00%	2.59%	0.00%
Strongly Agree	0.00%	1.72%	0.00%
No answer	0.00%	0.00%	0.00%

Question	Response count
2	366
9.4	369

9.5. My enjoyment of strength training is impactful in my ability to meet my goals:

My enjoyment of strength training is impactful in my ability to meet my goals:	What is the biological sex to which you were assigned at birth?		No answer
	Male	Female	
Strongly Disagree	0.00%	0.00%	0.00%
Disagree	0.75%	0.86%	0.00%
Neither Agree nor Disagree	6.72%	6.47%	0.00%
Agree	35.82%	34.48%	33.33%
Strongly Agree	56.72%	58.19%	66.67%
No answer	0.00%	0.00%	0.00%

Question	Response count
2	366
9.5	369

10.1. My strength training workouts are more frequent because of supervision by a personal trainer:

My strength training workouts are more frequent because of supervision by a personal trainer:	What is the biological sex to which you were assigned at birth?		No answer
	Male	Female	
Strongly Disagree	0.75%	1.29%	0.00%
Disagree	7.46%	5.60%	0.00%
Neither Agree nor Disagree	18.66%	17.67%	0.00%
Agree	31.34%	26.29%	66.67%
Strongly Agree	41.79%	49.14%	33.33%
No answer	0.00%	0.00%	0.00%

Question	Response count
2	366
10.1	369

10.2. My workouts would have similar frequency if I were not meeting a personal trainer:

My workouts would have similar frequency if I were not meeting a personal trainer:	What is the biological sex to which you were assigned at birth?		No answer
	Male	Female	
Strongly Disagree	23.13%	33.19%	66.67%
Disagree	47.76%	41.81%	0.00%
Neither Agree nor Disagree	14.18%	12.07%	33.33%
Agree	11.94%	9.48%	0.00%
Strongly Agree	2.99%	3.45%	0.00%
No answer	0.00%	0.00%	0.00%

Question	Response count
2	366
10.2	369

10.3. I value the accountability of meeting personal trainer for strength training workouts:

I value the accountability of meeting personal trainer for strength training workouts:	What is the biological sex to which you were assigned at birth?		No answer
	Male	Female	
Strongly Disagree	0.00%	0.43%	0.00%
Disagree	0.00%	0.00%	0.00%
Neither Agree nor Disagree	6.72%	3.45%	0.00%
Agree	32.84%	28.88%	66.67%
Strongly Agree	60.45%	67.24%	33.33%
No answer	0.00%	0.00%	0.00%

Question	Response count
2	366
10.3	369

10.4. I dislike having to schedule strength training workouts:

I dislike having to schedule strength training workouts:	What is the biological sex to which you were assigned at birth?		No answer
	Male	Female	
Strongly Disagree	38.06%	45.69%	0.00%
Disagree	45.52%	40.95%	66.67%
Neither Agree nor Disagree	10.45%	7.76%	33.33%
Agree	5.97%	4.31%	0.00%
Strongly Agree	0.00%	1.29%	0.00%
No answer	0.00%	0.00%	0.00%

Question	Response count
2	366
10.4	369

10.5. The regularity of my strength training is impactful in my ability to meet my goals:

The regularity of my strength training is impactful in my ability to meet my goals:	What is the biological sex to which you were assigned at birth?		No answer
	Male	Female	
Strongly Disagree	0.00%	0.43%	0.00%
Disagree	0.00%	0.43%	0.00%
Neither Agree nor Disagree	0.00%	0.86%	0.00%
Agree	26.12%	20.26%	33.33%
Strongly Agree	73.88%	78.02%	66.67%
No answer	0.00%	0.00%	0.00%

Question	Response count
2	366
10.5	369

11.1. My strength training workouts are safer because of supervision by a personal trainer:

My strength training workouts are safer because of supervision by a personal trainer:	What is the biological sex to which you were assigned at birth?		No answer
	Male	Female	
Strongly Disagree	0.00%	0.00%	0.00%
Disagree	0.00%	1.29%	0.00%
Neither Agree nor Disagree	6.72%	1.29%	0.00%
Agree	39.55%	21.98%	33.33%
Strongly Agree	53.73%	75.43%	66.67%
No answer	0.00%	0.00%	0.00%

Question	Response count
2	366
11.1	369

11.2. My workouts would be equally safe even if I were not supervised a personal trainer:

My workouts would be equally safe even if I were not supervised a personal trainer:	What is the biological sex to which you were assigned at birth?		No answer
	Male	Female	
Strongly Disagree	20.90%	34.05%	33.33%
Disagree	47.76%	44.40%	33.33%
Neither Agree nor Disagree	20.90%	14.22%	33.33%
Agree	9.70%	6.03%	0.00%
Strongly Agree	0.75%	1.29%	0.00%
No answer	0.00%	0.00%	0.00%

Question	Response count
2	366
11.2	369

11.3. I value the safety of having a personal trainer present for my strength training workouts:

I value the safety of having a personal trainer present for my strength training workouts:	What is the biological sex to which you were assigned at birth?		No answer
	Male	Female	
Strongly Disagree	0.00%	0.43%	0.00%
Disagree	1.49%	0.43%	0.00%
Neither Agree nor Disagree	11.19%	3.02%	33.33%
Agree	38.81%	28.88%	33.33%
Strongly Agree	48.51%	67.24%	33.33%
No answer	0.00%	0.00%	0.00%

Question	Response count
2	366
11.3	369

11.4. My strength training workouts are too focused on safety:

My strength training workouts are too focused on safety:	What is the biological sex to which you were assigned at birth?		No answer
	Male	Female	
Strongly Disagree	40.30%	49.57%	66.67%
Disagree	53.73%	41.81%	0.00%
Neither Agree nor Disagree	4.48%	6.90%	33.33%
Agree	1.49%	0.43%	0.00%
Strongly Agree	0.00%	1.29%	0.00%
No answer	0.00%	0.00%	0.00%

Question	Response count
2	366
11.4	369

11.5. The safety of my strength training is impactful in my ability to meet my goals:

The safety of my strength training is impactful in my ability to meet my goals:	What is the biological sex to which you were assigned at birth?		No answer
	Male	Female	
Strongly Disagree	0.00%	0.00%	0.00%
Disagree	3.73%	1.29%	0.00%
Neither Agree nor Disagree	10.45%	6.03%	33.33%
Agree	40.30%	29.31%	33.33%
Strongly Agree	45.52%	63.36%	33.33%
No answer	0.00%	0.00%	0.00%

Question	Response count
2	366
11.5	369

12.1. Supervision by a Personal Trainer optimizes strength training adaptations:

Supervision by a Personal Trainer optimizes strength training adaptations:	What is the biological sex to which you were assigned at birth?		No answer
	Male	Female	
Strongly Disagree	0.00%	0.00%	0.00%
Disagree	0.75%	0.00%	0.00%
Neither Agree nor Disagree	7.46%	3.45%	0.00%
Agree	32.84%	28.88%	33.33%
Strongly Agree	58.96%	67.67%	66.67%
No answer	0.00%	0.00%	0.00%

Question	Response count
2	366
12.1	369

12.2. Supervision by a Personal Trainer optimizes efficiency of a strength training workout:

Supervision by a Personal Trainer optimizes efficiency of a strength training workout:	What is the biological sex to which you were assigned at birth?		No answer
	Male	Female	
Strongly Disagree	0.00%	0.00%	0.00%
Disagree	0.00%	0.00%	0.00%
Neither Agree nor Disagree	1.49%	1.72%	0.00%
Agree	32.09%	21.98%	0.00%
Strongly Agree	66.42%	76.29%	100.00%
No answer	0.00%	0.00%	0.00%

Question	Response count
2	366
12.2	369

12.3. Supervision by a Personal Trainer reduces anxiety over performing strength training:

Supervision by a Personal Trainer reduces anxiety over performing strength training:	What is the biological sex to which you were assigned at birth?		No answer
	Male	Female	
Strongly Disagree	0.75%	2.16%	0.00%
Disagree	5.22%	5.60%	0.00%
Neither Agree nor Disagree	21.64%	12.93%	33.33%
Agree	40.30%	33.19%	0.00%
Strongly Agree	32.09%	46.12%	66.67%
No answer	0.00%	0.00%	0.00%

Question	Response count
2	366
12.3	369

12.4. Supervision by a Personal Trainer improves confidence when performing strength training:

Supervision by a Personal Trainer improves confidence when performing strength training:	What is the biological sex to which you were assigned at birth?		No answer
	Male	Female	
Strongly Disagree	0.75%	0.43%	0.00%
Disagree	0.75%	0.43%	0.00%
Neither Agree nor Disagree	8.21%	3.88%	33.33%
Agree	41.79%	31.90%	0.00%
Strongly Agree	48.51%	63.36%	66.67%
No answer	0.00%	0.00%	0.00%

Question	Response count
2	366
12.4	369

12.5. I have a more positive attitude toward strength training because of supervision by a personal trainer:

I have a more positive attitude toward strength training because of supervision by a personal trainer:	What is the biological sex to which you were assigned at birth?		No answer
	Male	Female	
**Strongly Disagree**	0.75%	0.00%	0.00%
**Disagree**	0.75%	3.02%	0.00%
**Neither Agree nor Disagree**	11.94%	7.76%	0.00%
**Agree**	28.36%	23.71%	66.67%
**Strongly Agree**	58.21%	65.52%	33.33%
**No answer**	0.00%	0.00%	0.00%

Question	Response count
**2**	366
**12.5**	369

12.6. Feedback from a personal trainer is an important part of my strength training:

Feedback from a personal trainer is an important part of my strength training:	What is the biological sex to which you were assigned at birth?		No answer
	Male	Female	
**Strongly Disagree**	0.00%	0.00%	0.00%
**Disagree**	0.00%	0.86%	0.00%
**Neither Agree nor Disagree**	3.73%	4.74%	0.00%
**Agree**	31.34%	31.03%	33.33%
**Strongly Agree**	64.93%	63.36%	66.67%
**No answer**	0.00%	0.00%	0.00%

Question	Response count
**2**	366
**12.6**	369

12.7. I have confidence in my personal trainer because of their experience:

I have confidence in my personal trainer because of their experience:	What is the biological sex to which you were assigned at birth?		No answer
	Male	Female	
Strongly Disagree	0.00%	0.00%	0.00%
Disagree	0.75%	0.43%	0.00%
Neither Agree nor Disagree	2.99%	3.02%	0.00%
Agree	33.58%	26.29%	33.33%
Strongly Agree	62.69%	70.26%	66.67%
No answer	0.00%	0.00%	0.00%

Question	Response count
2	366
12.7	369

12.8. I have confidence in my personal trainer because of their qualifications:

I have confidence in my personal trainer because of their qualifications:	What is the biological sex to which you were assigned at birth?		No answer
	Male	Female	
Strongly Disagree	0.00%	0.00%	0.00%
Disagree	0.00%	0.86%	0.00%
Neither Agree nor Disagree	5.97%	3.45%	0.00%
Agree	46.27%	31.90%	33.33%
Strongly Agree	47.76%	63.79%	66.67%
No answer	0.00%	0.00%	0.00%

Question	Response count
2	366
12.8	369

12.9. I enjoy working with a team of different personal trainers:

I enjoy working with a team of different personal trainers:	What is the biological sex to which you were assigned at birth?		No answer
	Male	Female	
**Strongly Disagree**	0.75%	3.02%	0.00%
**Disagree**	11.94%	9.48%	33.33%
**Neither Agree nor Disagree**	17.91%	22.41%	33.33%
**Agree**	36.57%	34.05%	0.00%
**Strongly Agree**	32.84%	31.03%	33.33%
**No answer**	0.00%	0.00%	0.00%

Question	Response count
**2**	366
**12.9**	369

Female

Response	Coding
1. In the past I attempted strength training without a trainer. It was not successful, and I didn't keep at it. I appreciate the relationships I form with my trainers.	Personal relationship<br>Accountability
2. I appreciate the focused 30 minutes for efficient strength training workouts that i don't need to think about.	Being told what to do – Knowledge/program Design
3. If I were strength training myself, it would be a lot easier to skip workouts (I've done it in the past). Having an appointment with my strength trainers ensures that I make it a priority and follow through with the workout. Also, if I hurt a certain area of my body (for example, strained my neck), I usually would avoid working out altogether. Being able to tell my strength trainer how I'm feeling or where it hurts is very helpful in still being able to get a workout in while avoiding hurting myself further.	Accountability<br>Program Design
4. It has changed my life in a positive way. I'm more consistent and feel safe and motivated to continue	Accountability<br>Safety
5. I love the trainers at Discover Strength! They are so knowledgeable and friendly (special shout out to Sophie at North East!!!)	Personal relationship, knowledge/program design
6. The knowledge shared by the trainers about specific benefits of strength training, why certain techniques and exercises are used, the science behind techniques is also a key benefit of training with DS trainers	knowledge/program design
7. Discover Strength in Chanhassen, MN (USA) has improved my husband and my lives beyond our expectations. I feel blessed to have DS in our community.	Adaptations
8. Basically, I wouldn't strength train if I didn't have a trainer. I trust their knowledge and experience and I'm happy with progress I've made.	Accountability, knowledge/program design
9. After a lifetime of failing to achieve my fitness goals through various programs, I have finally achieved a high degree of satisfaction with the results.	Adaptations
10. Strength training with a trainer at Discover Strength is the first time I have been able to stick to a strength training program and have seen tangible results.	Accountability<br>Adaptations
11. As I have aged, I appreciate my trainer's ability and knowledge in creating challenging programs and pushes me to continue to improve.	Knowledge/program design
12. It provides efficiency that is needed in a busy lifestyle. More variety of exercises has been helpful. I do virtual training sessions which I love since I live in a very rural area. I did not	Time efficiency<br>Program design<br>Adaptations

expect having noticeably more energy throughout my day and enjoy just moving.	
13. I've particularly appreciated my trainer's modification of my training when an injury occurs. (Injury was NOT from training)	Knowledge/program design
14. Previously I have not been able to stick with a strength training program. It is like night and day. I have been going to Discover Strength for over a year now and I love it. I feel better and I look forward to going every time. It is definitely a motivator for me to work with a trainer.	Accountability<br>Motivation/enjoyment
15. Strength training kept me strong during 9 months of cancer treatment now 5 years ago, and I continue to make progress in building strength at age 68.	Adaptations (ill-health)
16. At the age of 71 I completed my 10th Cross country skiing marathon this year, after ankle replacement 9 months earlier. This is possible only that I have used supervised strength training with Discover Strength since 2010 1-2x per week without fail. My ankle doctor said I was a month or more ahead of others in my recovery due to my strength and commitment due to supervised strength training	Adaptations
17. I like the accountability of supervised strength training and that they keep track of my progress and increase my weight depending on my progress. I also like them correcting my form. But I don't like the rigidity of the workouts, for example that you are not allowed to add unsupervised easy activities such as lunges or band walks or planks at the end of the workout if your time is up.	Accountability<br>Program design<br>Technique/form<br><br>DISLIKE – rigidity of workouts (autonomy?)
18. Provides accountability for me with calls and texts checking in on me when I "take a break" and this kicks me in the behind to go back to workout again.	Accountability
19. Have more diverse trainers at DS (women, young and older, different social economic backgrounds, racial diversity) tired of just training with young white men. They're super polite, knowledgeable about strength training. But don't get aging bodies, aging goals and especially aging women's bodies. Why aren't there a few older (over 40 which isn't old!) women trainers.	Relationship with trainers (diversity)<br>Knowledge
20. I love being pushed to my limits and I don't think I would do that without somebody else pushing me! The accountability is also important as well as well as the efficiency it allows.	Effort<br>Accountability
21. I would love to have a summarized recap of feedback after each session. Almost like an area of improvement or how to improve if I struggled in a certain movement or sequence.	Feedback
22. I prefer to work with the same personal trainer all the time... you are on a self-improvement journey, so if you can have the	Personal relationship

same person vested in your journey, I think you will have better results overall in the personal training experience...	
23. Work with a trainer on a consistent basis builds my trust and confidence	Confidence
24. I have really valued my time strength training under supervision. I know how to do cardio but strength always intimidated me. I didn't know how best to create a workout, keep myself motivated if I wasn't in the mood to finish a rep or even if I was safely performing a workout. I could do some workouts, but I wasn't confident that they were even effective. I've been doing strength training now for almost 4 years and I have noticed change, it's great!	Knowledge/program design<br>Accountability<br>Accountability
25. Couple of points - I do like training with different trainers, but a handful only. I think it's really important for the trainer to know you. However, it also is good to have 3-4 different ones because they do things different. Also, I have been going to DS for 11 years. I can't imagine not going. My original goal was to maintain my strength....and it's increased even though I'm 65 years old now. It also makes me accountable for going to the studio (or virtual). I love Discover. Luke has done a great job with the culture that exists with the trainers. They are very respectful, fun, and good at what they do.	Personal relationship<br>Accountability
26. Personality compatibility with my trainer is Extremely important to me I need to be in charge of who I with.	Personal relationship
27. I work for praise and believe every encouraging comment I get from my trainers. I would not strength train at all if I did not have trainers who encourage me.	Motivation / enjoyment<br>FEEDBACK<br>Accountability
28. I have participated in supervised strength training for the past 18 yrs. I definitely started as a way to compliment and improve my running and quickly discovered that it was more important to my long term health than anything else I do. Supervision has allowed me to strength train at a level I never would have thought I was capable of on my own. And I know that I would have stuck with it this long on my own. The challenges, encouragement, successes, and results that I celebrate with my trainer(s) keeps me coming back week after week and year after year.	Accountability<br>Adaptation
29. I wouldn't be doing strength training if I had to do it on my own. My trainer Kyle L at SLP is the reason I am sticking with it and enjoying it!!	Accountability
30. I like a human trainer because they're attentive to any nonverbal cues or idiosyncrasies like my form or energy level/mood. On another. note, I also understand it's a privilege to afford a personal trainer and be able to work out so often.	Personal relationship

31. I do it to get the correct weights and exercises to maintain/increase my strength. I appreciate them setting the standard/expectation and correct my technique - but do not need my trainer to motivate me with positive feedback or encouragement. Just tell me what is expected and make sure I do it right.	Program design<br>Technique/form
32. I like the continuity of having the same trainer rather than, as is often the case, getting a rotation of different and new trainers	Personal relationship
33. The trainers are great. Professional, friendly and really motivating DS is a very welcoming place	Personal relationship<br>Motivation/enjoyment
34. There is absolutely no way I would have made the progress I have without my sessions being supervised.	Adaptations
35. I don't like it when a trainer stands with me and counts. I'd rather have a conversation while they time me or keep track of repetitions in a different way.	Personal relationship
36. Supervised strength training adds a layer of accountability to the workout. You not only have a scheduled time, but also a scheduled guide (in your supervisor/coach). With this, it allows workouts to more easily become a “sticking habit”- you are showing up to each workout for more than the appointment; you are showing up for the guidance and motivation from your supervisor.	Accountability
37. I love the supervised strength training. Form is important and pushing a person past their comfort zone is important. I have become very attached to certain trainers just like a dentist or doctor. DS needs to not just switch with different trainers than what a customer signs up for without the customers approval. This is an issue that needs to be addressed.	Technique/form<br>Effort<br>Personal relationship
38. I enjoy the structure of being able to schedule it for a convenient time and with DS, I enjoy the time economy of the 30 min workout. Music is also key - bumping music is a nice motivator.	Accountability
39. I have seen how the twelve years of strength trading at DS has had an impact on my abilities to perform tasks without fear of injury. I've learned so much about how strength training affects me on so many levels. I have been made to feel that the trainers I have had really care about me as a person and are always so positive and encouraging!	Program design<br>Personal relationship
40. My personal trainer listens to my physical issues (plantar fasciitis, weak low back) and makes adjustments accordingly. I value this!	Program design
41. I would not do regular strength training without a personal trainer. I very much value everything about working with a trainer.	Accountability

42. I would not lift as heavy or go to failure without having someone push me and make me accountable	effort
43. I have been strength training at Discover Strength for over 5 years. I find it incredibly important to strength train with the same trainer because he/she knows my history and what exercises can cause an unpleasant outcome. I do not like to explain my history to multiple trainer's, I find that to be stressful and time consuming. I also do not care for trainer's who do not listen to my input, with regards to my workout program, into consideration. I know my body and what I can and cannot do that will impact me positively or negatively. I think that is an important takeaway, that what or how the trainer's train themselves is not always a good fit for the client and it ought to be a open discussion, not a you're going to do this without considering the individual.	Personal relationship<br>Program design
44. I have consistently been working out twice per week, both in studio and virtual for over 17 years. I am very healthy and was able to recover from.a slight meniscus tear without surgery through supervised strength training. I am a huge proponent of supervised strength training to slow the aging process physically and mentally!	Adaptation<br>accountability
45. I had a significant number of years of personal strength training prior to joining Discover Strength (DS), and I have had relatively good form and discipline going into training with them. Having said that, I find that the DS help me further improve my form (and adapt workouts to various aches and pains) such that the quality of my workouts is even greater.	Technique/form<br>Programme design
46. Motivation, feedback on form, my confidence in training vary from trainer to trainer.	Motivation/Enjoyment<br>technique/form
47. When I started at DS over 2.5 years ago, my plan was six months of supervised strength training and then do it on my own (as I had done in years past). However, after I experienced the benefits, I decided that my health was worth the investment. The DS philosophy and techniques hit the sweet spot at 2x per week and are foundational for keeping me physically, emotionally, and mentally strong. Great team at Woodbury, MN!	Adaptations
48. When I am dealing with an injury, having supervised training is a must. I came down with frozen shoulder. Without the supervision and expertise along with the endless motivation, I would have not trained through the frozen shoulder, I would have stopped training.	Program design /<br>knowledge
49. energy and full attention is critical in a trainer; communication is key and understanding my goals and limitations	Programme design

50. Having a background in athletics I recognize the importance of strength training. It is motivating, rewarding and enjoyable to have supervised weight training bc the value it has for me mentally and physically today but also as I age.	Motivation/enjoyment<br>Adaptations
51. Pushes me beyond what I thought I could do, as well as encourages regularity in strength training.	Effort<br>Accountability
52. It took longer than I anticipated to see results, however, when they came, THEY CAME.	Adaptations
53. I have tried to quit a few times and always come back to it! I have healed multiple injuries through strength training with supervision. Now, we just need health insurance providers to see the value and cover some or all of it :)	Adaptations
54. My goals are to have fun and consistently strength train for long term health which influences my answers. I don't value getting stronger, better, technique as much as the trainers do. It feels good but it equally feels good to have the consistency of a strength workout every week for years. While I know the business needs the staff to grow and turnover, I appreciate the trainers that I have built a relationship with more than having to do it over and over again. But it doesn't keep me from scheduling with different people. I just enjoy the workouts better with my regular trainer. It takes about 3 months with a new person before I am not disappointed that my favorite trainer is gone. And probably another 3 months before they become my favorite trainer. Then about 6 months later something changes and I am back to a new person.	Adaptations<br>Accountability<br>Personal relationship
55. I really enjoy strength training through DS. Best training results I've ever had.	Adaptations
56. Note: I'm a virtual client at Discover Strength, but I still feel my workouts are very effective. Thanks to Alex Cizek and Maria Hauger for being awesome trainers!	Adaptations
57. Strength training with discover strength is one of the best things I do for myself, and I look forward to every session because of the positive, professional, welcoming, motivating, and encouraging atmosphere provided by the exceptional staff.	Professional<br>Motivation/enjoyment
58. Absolutely love and respect my trainers at DS. They motivate and encourage me when I'm down or less than confident. It matters. I have achieved gains because of their expertise.	Effort<br>Motivation/enjoyment
59. While it is for health and fitness reasons that I've added strength training to my workout, a big reason is also related to perimenopause and the trend for women to do more strength training. I don't always feel like the trainers understand the science of menopause for women, the changes in body shape and hormone levels and benefits of strength training. With question #1 - I didn't feel like women's change in muscle/body	Adaptations

shape and hormones during peri/menopause was included in the provided answer options.	
60. My opinion on input from personal trainers is based upon the ones I work with. I have observed two others at DS who are over the top in their verbal encouragement (in volume and attitude) and I would not have stayed with DS were they my regular trainers.	NEGATIVE – personal relationships
61. working out with a trainer has been a huge factor in my consistency over the years.	Accountability
62. I started strength training knowing nothing about weights, technique, or best practices. I would not be strength training without the supervision and assistance of a personal trainer.	Knowledge/program design<br>Accountability
63. Without personal supervision the progression of muscle strength would not have a forward movement, but stagnation. Being pushed by a personal trainer truly encourages one to push themselves harder without the worry of injury. Plus, it's great for mental stimulation.	Adaptations<br>Effort
64. My feedback may be skewed by my training as a physical therapist. Personally, I need the accountability of supervised strength training more than feedback on technique/form. But for many people, I think feedback on technique/form is integral for strength training to decrease risk of injury and promote strength and longevity	Accountability<br>Technique/form
65. I gained even more appreciation for supervised training after my 2022 stroke. It is only by working with my DS trainers that I have gained back as much as I have in the past two year.	Adaptations
66. DS has been life changing for me. I started 4 or so years ago with you, and the accountability, the professionalism, the engagement, the push, fair-but-firm approach to my goals ... I'm hooked. I feel stronger at 62 than I ever have, and also feel more capable and healthier because of this than ever before. Total advocate for you guys...and I love that the trainers are all educated...this makes a huge difference.	Adaptations<br>Knowledge – education
67. I love my DS workouts and the more active the trainer is on all the factors you asked about, the more beneficial and enjoyable I find the work.	All characteristics
68. Benefits can be very trainer specific. Also - some trainers don't realize that different clients need to be motivated/pushed to different extents - one size does not fit all	Personal relationship

Male

Response	Coding
1. I have gained significantly more strength and muscle size after training with a personal trainer for 3 years.	Adaptation
2. Having a trainer brings a level of confidence when working out. They work the areas the right way and encourage you to fight through the discomfort, I like being able to walk in be told what to do.	Confidence<br>Effort<br>Being told what to do – Knowledge/program Design
3. Simply put, without being accountable to a trainer, I would not be as motivated.	Accountability
4. The question about frequency of workouts being more impacted, with a trainer, misses a more important point to examine…what is the LONG-TERM frequency influence, of a trainer? When left to my own, I'll go into the gym more frequently and spend more time (to get to some pre-set goal). Once I hit that goal (or injure myself), I'm done and am back into no workout mode. Because of the partnership with personal trainers, I'm closing in on one full year of consistent workouts. The last time I continued workouts on my own, for that long, was when I was a teenager. Most importantly I know that as long as I can stay healthy and budget for it, I can and desire keep this going for many more years.	Long-term accountability, based on continuing even after achieving a desired goal
5. The close supervision and extra reps the trainers help you safely perform are very impactful!	Safety
6. Much more focused with personal trainer that gets to know you and your goals and reasons for strength training.	Personal relationship
7. Having an appointment system is most advantageous to me for accountability. I wasn't personally accountable enough to do it on my own.	Accountability
8. In the past I did strength training on my own. I certainly did not progress like I have since using a personal trainer. I did not have the knowledge to make changes as necessary, and having somebody to push me to get the most out of my workouts is an important aspect of supervised strength training to me.	Accountability<br>Program Design / Knowledge<br>Effort
9. Wouldn't be nearly as consistent without regularly scheduled supervised training	Accountability
10. Having a strength training appointment helps hold me accountable and keeps me working out consistently.	Accountability
11. Repeating what much of this survey already says, the overall quality, frequency, consistency of my workouts	Accountability

is enhanced by having scheduled workouts with a personal trainer. I like being able to schedule weeks in advance and the ease of the scheduling process.	
12. I could never do unsupervised strength training on a regular basis anymore.	Accountability
13. Supervision is essential to my successful training.	Adaptations
14. Value the trainer's ability/expertise in modifying workouts to accommodate injuries, aches/pains…	Program Design
15. To some extent, there is also a monetary motivation to scheduled workouts with trainers. I'm less likely to skip a workout because of the accountability to the trainer, but also a mentality of "I've already paid for the workout, so I really need to go."	Financial commitment enhances accountability
16. For me, having someone help with a clear plan and process provides consistent results while saving time working out.	Program Design
17. The accountability, professionalism, and encouragement from ALL the trainers at DS is what keeps me coming back.	Accountability<br>Effort / Motivation – FEEDBACK<br>Professionalism
18. All ratings are based on the assumption of a quality personal trainer. A poor one can be more detrimental than doing it on your own. In terms of the question around value of working with a team, I love the ability to work with a cohesive team with the ability to have a primary person who really gets to know me as I am able to do at Discover Strength.	Personal relationship / Professionalism
19. I feel like I'm not going to injure myself with supervision which is a huge consideration given time off to recover from an injury.	Safety
20. Maintaining the high standard of trainers is important to maintaining the quality of the experience.	Quality of trainer / Professionalism
21. Trainers need to be flexible and adapt the training to changes in the client from injury or health issues	Program Design
22. I like having 2-3 specific trainers I work with. It's important because they learn me. My strengths. My weaknesses. My needs. My personality. Having too many trainers to have to work with is difficult as I need to train them on how to train me.	Personal relationship
23. As someone who didn't strength train before, the perspective, enthusiasm, and attitude of the person supervising my strength training has had an influence on me.	Personal relationship
24. The trainers at DS have a superb work ethic and professionalism in addition to being extremely	Program Design<br>Professionalism

knowledgeable about high intensity weight training. They are a highly effective group.	
25. I had the experience of trying strength training several times without supervision prior to a beginning to train with supervision 9 years ago, so that helps clarify its importance to me.	Benefits of supervision compared to unsupervised
26. I have enjoyed all the trainers I have worked with over the year I have been training at the Woodbury, MN location.	Motivation / enjoyment
27. As a 75-year-old male, I strongly believe that without regular strength training, my quality of life would not be what it is today.	Adaptations
28. The personal relationship with the trainer is a significant factor. I maintain/have maintained supervised workouts for 5 years because of the personable and engaging people.	Personal relationship
29. I have gained significantly more strength and muscle size after training with a personal trainer for 3 years.	Adaptations
